# Orthopedic surgery residents’ perception of online education in their programs during the COVID-19 pandemic: should it be maintained after the crisis?

**DOI:** 10.1080/17453674.2020.1776461

**Published:** 2020-06-15

**Authors:** Francisco Figueroa, David Figueroa, Rafael Calvo-Mena, Felipe Narvaez, Natalia Medina, Juan Prieto

**Affiliations:** a Clinica Alemana-Universidad del Desarrollo, Santiago;; b Hospital Sotero del Rio, Santiago;; c Pontificia Universidad Catolica, Santiago;; d Universidad de Santiago, Santiago;; e Universidad de Chile, Santiago, Chile

## Abstract

Background and purpose — During the COVID-19 pandemic, most of the teaching centers in Chile have shifted to online resources. We decided to do a survey on orthopedic residents regarding this type of education to assess for strengths and weaknesses of digital education in orthopedic programs.

Methods — A survey was performed targeting 110 orthopedic residents belonging to different training programs around the country. 100 residents completed the survey.

Results — 86% stated that their programs are using online education. When asked in detail, 86% had been involved in webinars, 28% had received online presentations, 12% had participated in online tests, and 7% had evaluated patients. Webinars were rated (1 = very unsatisfactory, 10 = very satisfactory) with a mean grade of 8.1 (1–10), online presentations 7.3 (1–10), online tests 3.8 (1–8), and online patient evaluations 2.9 (1–9). When asked if, after the end of the pandemic, they would continue using the online modalities, 82% would continue attending webinars, 72% would continue watching online presentations, 27% would continue performing online tests, and 33% of the residents would continue performing online evaluations of patients.

Interpretation — Even though resident evaluation of online activities is positive, face-to-face theoretical activities are still valued as a necessary complement for orthopedic residency education.

The rapidly unfolding pandemic COVID-19 implies a surge in inpatient demand that may exceed the capacity of health systems even in developed economies (Ministry of Health Chile 2020). In response, local health governances have adopted drastic changes to care structures, particularly the operating theatre, deferring and cancelling elective surgeries, and limiting their functioning to urgent cases (Ministry of Health Chile 2020). On the other side, the population has been recommended to stay at home, with some locations including government-regulated quarantine in an attempt to limit the propagation of the disease. One of the results of these measures has been a dramatic decrease in outpatient and inpatient activity for orthopedic surgeons and their trainees, including a drastic reduction in the amount of surgeries. Restrictions of academic meetings and departmental gatherings among others have also been part of the efforts made to limit the spread of the virus and its impact on health workers. A regular day in orthopedic training includes patient rounds, surgeries, seminars, and research activities, all of which have been affected by the health ministry restrictions.

In a response to this situation, and in an attempt to maintain a reasonable number of educational instances for residents, our department and most of the teaching centers in Chile have shifted to online resources to continue the education of our residents. Online learning or e-learning is the use of internet-based resources for teaching and learning (Jayakumar et al. 2015). The advantages of e-learning in the surgical setting are well established. In addition to being easily accessed and updated, e-learning platforms accommodate a wide variety of learning styles and can effectively teach a broad array of surgically relevant information (Tarpada et al. 2016).

A number of studies indicate that both surgical residents and medical students who use e-learning programs are more satisfied with their learning experience (time invested for learning in relation to learning they obtained, organization and clarity of the study subjects, and better performance on simulated cases, among others) compared with those who use traditional teaching methods (Citak et al. 2009, Horstmann et al. 2009, Funke et al. 2013). While use of e-learning platforms in surgical training has expanded over recent years, the role of this technology within orthopedic surgery residents remains scarcely studied. Tarpada et al. (2016) in a recent systematic review found 3 studies (Ceponis et al. 2007, Hearty et al. 2013, Heiland et al. 2014) regarding orthopedic residents’ online training for different surgical skills, all with promising results including better performance at diagnostic shoulder arthroscopy, better knowledge of the technique for closed reduction and pinning of supracondylar humeral fractures, and better surgical skills in spine surgery.

Our personal thought is that this modality of education has arrived abruptly in the different orthopedic residency programs, but has arrived to stay. Therefore, we decided to conduct a survey among orthopedic residents on a broad range of teaching programs in Chile to identify the strengths and weaknesses of this form of transmitting knowledge for the future of the orthopedic surgery.

## Methods

A survey was sent to 110 orthopedic residents in the first, second, and third year of education, who were on 7 different training programs around Chile on April 13, 2020. The survey was developed using the SurveyMonkey software (Palo Alto, CA, USA) and sent via WhatsApp (Mountain View, CA, USA). Residents were informed about the nature of the survey and that the results were to be used as part of a study. All the participants gave formal consent to be included in the analysis. The results were collected 5 days after the survey was sent. 100 residents answered the survey (80 male) with an age range from 25 to 34 years, representing around 50% of the total number of residents in Chile (Table).

## Conflicts of interest

The authors declare no conflicts of interest related to the subject of this study.

## Results

**Figure 1.**
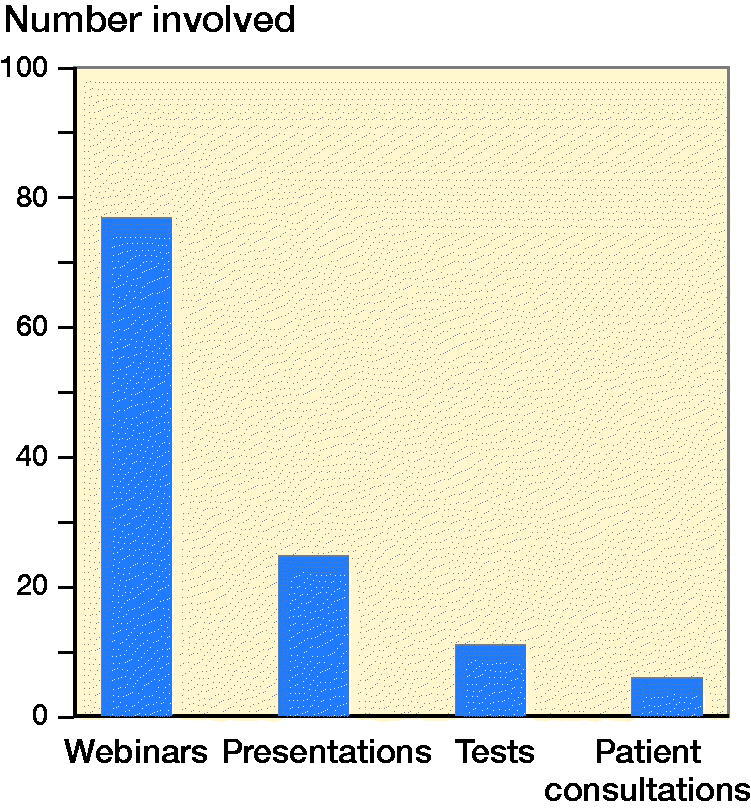
Number of residents involved in different online modalities.

Regarding the utilization of online instances to compensate for the decrease in formal face-to-face training, 86% of the residents stated that their programs are using this type of education. When asked in detail about the specific modalities used, the most common was online seminars with the opportunity to discuss topics from the audience (webinars) (Figure 1). Other options mentioned by the residents were: online presentations, online evaluations or tests, and video patient consultations. The main app that has been used is “Zoom” (Zoom Video Communications, San Jose, CA, USA), in 88% of cases.

**Figure 2.**
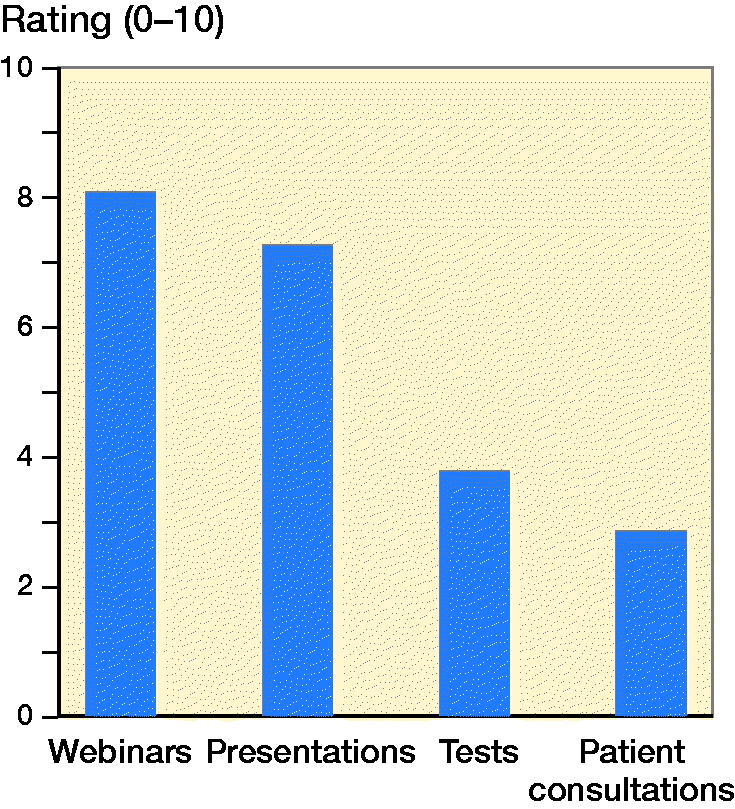
Evaluation of the different online modalities.

Regarding how the residents rate the different online learning experiences grading from 1 to 10 (with 1 being very unsatisfactory and 10 being very satisfactory), webinars were rated highest with a mean grade of 8.1 (Figure 2).

The most important difficulties of the different modalities for online education mentioned by the residents were: technical aspects (slow internet connection, audio problems) for 42% of the residents, absence of practical education in surgical training for 13%, lack of concentration due to home distractions for 9%, and difficulties with scheduling and overload of seminars/presentations for 9%.

**Figure 3.**
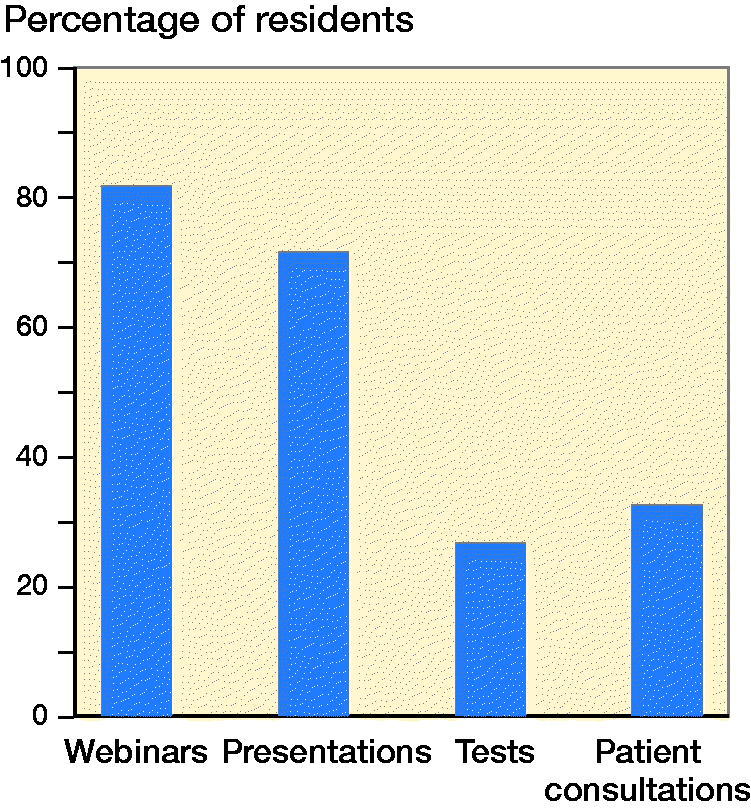
Percentage of residents who would continue with different online modalities.

When asked if, after the end of the pandemic, they would continue using the online modalities performed in the last weeks, most of the residents answered that they would continue attending webinars and would continue to perform online presentations. On the other hand, only a minority would continue performing online evaluations and tests and performing video consultations with patients (Figure 3).

30% of the residents believed that, when the COVID-19 pandemic is over, all non-practical medical education should be performed using internet-based tools. Similarly, 40% of the residents responded that academic meetings (orthopedic department meetings for example) or meetings involving less than 100 participants, should be done in a non-face-to-face fashion.

## Discussion

As the COVID-19 pandemic spreads around the world (World Health Organization 2020), and with the future of social interaction already unknown, orthopedic residency programs must consider different paths of learning. Meetings and conferences that previously took place face-to-face will now forcibly be done using internet-based spaces for an unknown period of time. However, despite the difficulties associated with this change of paradigm, human minds always flourish and solutions are found to new problems. Weeks ago, we would have never thought that clinical meetings or even complete congresses would be completely undertaken using online resources, without the need to move from one’s home or office. As this is new to residents in Chile, and they are the primary objective of these measures, we decided to survey them to learn about their preferences and the strengths and weakness of the different teaching methods during this pandemic, considering which should survive after the pandemic is over, and which should disappear.

We found notable preferences; for example, it seems that webinars and any kind of online presentation with the possibility for the audience to participate have arrived to stay with excellent ratings among residents. In addition, online presentations, a resource that was widely used around the world before the pandemic, are reinforced as an excellent teaching method because of the chance of being widely distributed with the resources already available today. Conversely, online tests or exams and video patient consultations seem to be only contingent solutions for a momentary problem rather than a permanent modality of teaching, with both being widely rejected by orthopedic residents around the country.

Despite belonging to the OECD (Organization for Economic Cooperation and Development) (OECD 2020) and having the 4th fastest internet speed in America (Fastmetrics 2020), technical problems are still important during online activities that require more data transmission than the regular amount, with loss of connection or delay and decrease of fluency reported. Technical problems are frequently reported in articles evaluating online education methods in medicine, with one-quarter of the participants in the study by Horstmann et al. (2009) reporting this issue. The same problem is listed in the Jayakumar et al. (2015) systematic review on e-learning for surgical education. Eventually, with the dramatic increase in virtual meeting applications (Digital Trends 2020) because of the pandemic, there will be advances in the consumption of data required, and this problem will be solved.

Another challenge noted was the necessity to obtain an adequate environment in the resident’s home, avoiding the many distractions that usually are not present in a more formal situation. Additionally, scheduling and overload of online activities were mentioned as a recurrent problem for residents. Regular training activities in Chile during normal conditions are a mixture of more practical activities (patient rounds, surgeries) than academic theoretical training. However, because of the COVID-19 pandemic, and the decrease in orthopedic surgery activity, an important number of trainers now have more time to perform seminars, webinars, or other modalities of online training. This produces a sense of overload in the residents and scheduling issues (more than 1 activity at the same time). The solution to this problem involves better coordination and communication between trainers and the ability to provide a formal schedule of activities at reasonable times.

Finally, in spite of the good evaluation of some of the online modalities, as noted in the final 2 questions, most of the orthopedic residents believe that after the pandemic is over non-practical medical education should be at least in part face-to-face instead of being completely online. This corresponds to the results presented by Funke et al. (2013) in which after a questionnaire to evaluate an online teaching method, teaching in small groups and face-to-face learning obtained the best scores compared with online modalities. This creates a challenge for educators, because they need to balance online and in-person modalities, as face-to-face theoretical activities are still valued by most residents as a necessary complement to their education as orthopedic surgeons.

In summary, in an attempt to maintain a reasonable number of educational instances for residents, most of the teaching centers in Chile have shifted to online resources. Even though the evaluation of some of the activities is good (webinars, presentations), face-to-face theoretical activities are still valued as a necessary complement for their education as orthopedic surgeons.

**Table:** Full questionnaire with residents’ answers

		Rating
	n	mean (range)
1. Has your program started any internet-based education modalities because of the COVID-19 pandemic? (If your answer is yes go to question number 2, if your answer is no go to question number 7)		
• Yes	86	
• No	14	
2. In what type of online modalities have you participated? (		
You can mark more than 1 answer)		
• Webinars (seminars with discussion		
and participation of the audience)	77	
• Presentations	25	
• Surgical videos	0	
• Exams and tests	11	
• Video consultation on real patients	6	
• Other	0	
3. What web apps have you used for these instances?		
• Zoom	78	
• Microsoft Teams	8	
4. How do you rate the online education modalities received during the COVID-19 pandemic? (Grades from 1 to 10, 1 is very unsatisfactory, 10 is very satisfactory)		
• Webinars		8.1 (1–10)
• Presentations		7.3 (1–10)
• Surgical videos		Not rated
• Exams and tests		3.8 (1–8)
• Video consultation on real patients		2.9 (1–9)
• Other	Not rated	
5. What difficulties do you see with this form of education?		
(Free text question)		
• Technical aspects (slow internet		
connection, audio problems)	36	
• Absence of practical education	11	
• Lack of concentration due to home		
distractions	8	
• Difficulties with scheduling and overload		
of seminars/presentations	8	
6. After the pandemic is over, which online-based educational instances would you continue to use?		
(You can mark more than 1 answer)		
• Webinars	63	
• Presentations	18	
• Surgical videos	0	
• Exams and tests	3	
• Video consultation on real patients	2	
• Other	0	
7. Do you believe that after the pandemic is over, all the theoretical education of orthopedic residencies should be done using web-based platforms?		
• Yes	30	
• No	70	
8. Do you consider that educational instances with groups smaller than 100 people (orthopedic department meetings, small subspecialist meetings) should be done using web-based platforms after the pandemic is over?		
• Yes	40	
• No	60	
